# Update: Ebola Virus Disease Epidemic — West Africa, December 2014

**Published:** 2014-12-19

**Authors:** Incident Management System Ebola Epidemiology Team, CDC; Guinea Interministerial Committee for Response Against the Ebola Virus; World Health Organization; CDC Guinea Response Team; Liberia Ministry of Health and Social Welfare; CDC Liberia Response Team; Sierra Leone Ministry of Health and Sanitation; CDC Sierra Leone Response Team; Viral Special Pathogens Branch, National Center for Emerging and Zoonotic Infectious Diseases, CDC

CDC is assisting ministries of health and working with other organizations to end the ongoing epidemic of Ebola virus disease (Ebola) in West Africa (1). The updated data in this report were compiled from situation reports from the Guinea Interministerial Committee for Response Against the Ebola Virus, the World Health Organization, the Liberia Ministry of Health and Social Welfare, and the Sierra Leone Ministry of Health and Sanitation. Total case counts include all suspected, probable, and confirmed cases, which are defined similarly by each country (2). These data reflect reported cases, which make up an unknown proportion of all cases, and reporting delays that vary from country to country.

According to the latest World Health Organization update on December 10, 2014 (3), a total of 17,908 Ebola cases have been reported as of December 7 from three West African countries (Guinea, Liberia, and Sierra Leone) where transmission is widespread and intense. The highest reported case counts were from Sierra Leone (7,897cases) and Liberia (7,719), followed by Guinea (2,292). Peaks in the number of new cases occurred in Liberia (509 cases), Sierra Leone (748 cases), and Guinea (292 cases) at epidemiologic weeks 38 (September 14–20), 46 (November 9–15), and 41 (October 5–11), respectively (Figures 1 and 2). A total of 6,373 deaths have been reported. Investigation of localized transmission in two locations in Mali (Kourémalé and Bamako) is ongoing, with a current total of eight cases and six deaths reported (4). Transmission was interrupted successfully in Nigeria (October 19) and prevented in Senegal (October 17) (3).

There were 4,281 new Ebola cases reported during the 4-week period of November 9–December 6, compared with the 2,705 new cases reported during the 3-week period of October 19–November 8 (5). Cases were widely distributed geographically among districts in all three countries, with the prefecture of Mamou in Guinea reported to be newly affected. During both periods, counts of reported Ebola cases were highest in the area around Monrovia, including Grand Cape Mount, Liberia; the Western Area and northwest districts of Sierra Leone, particularly Bombali and Port Loko; and Conakry, Guinea (Figure 3).

As of December 6, the highest cumulative incidence rates (>100 cases per 100,000 population) were reported by two prefectures in Guinea (Guéckédou and Macenta), six counties in Liberia (Bong, Grand Cape Mount, Lofa, and, particularly, Bomi, Margibi, and Montserrado, with cumulative incidence of >300 cases per 100,000 population), and six districts in Sierra Leone (Bombali, Kailahun, Kenema, Port Loko, Tonkolili, and Western Area) (Figure 4). Evidence of decreasing incidence in Lofa and Montserrado, Liberia, has been described elsewhere (6–8), though cases continue to be reported from these counties, especially Montserrado.

The latest updates on the 2014 Ebola epidemic in West Africa, including case counts, are available at http://www.cdc.gov/vhf/ebola/outbreaks/2014-west-africa/index.html. The most up-to-date infection control and clinical guidelines on the 2014 Ebola epidemic in West Africa are available at http://www.cdc.gov/vhf/ebola/hcp/index.html.

## Figures and Tables

**FIGURE 1:**
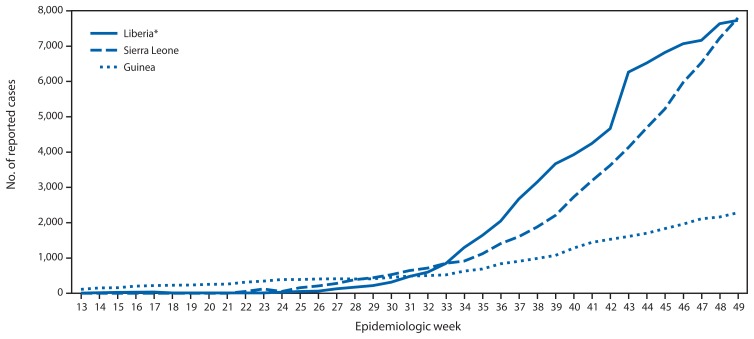
Cumulative number of Ebola virus disease cases reported, by epidemiologic week — three countries, West Africa, March 29–November 30, 2014 * A change in reporting source data at week 43 resulted in an adjustment of cumulative cases in Liberia.

**FIGURE 2:**
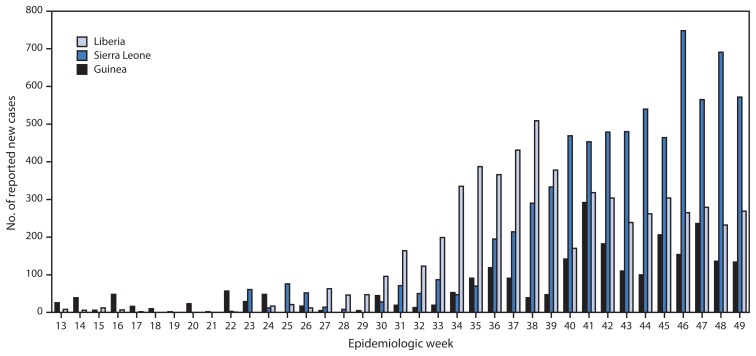
Number of new Ebola virus disease cases reported, by epidemiologic week — three countries, West Africa, March 29–November 30, 2014

**FIGURE 3:**
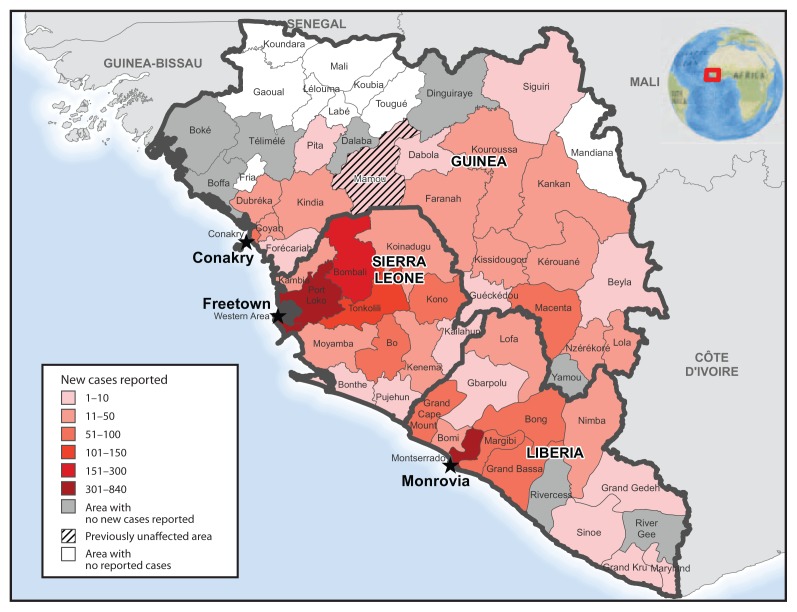
Number of new cases of Ebola virus disease reported — Guinea, Liberia, and Sierra Leone, November 9–30, 2014

**FIGURE 4:**
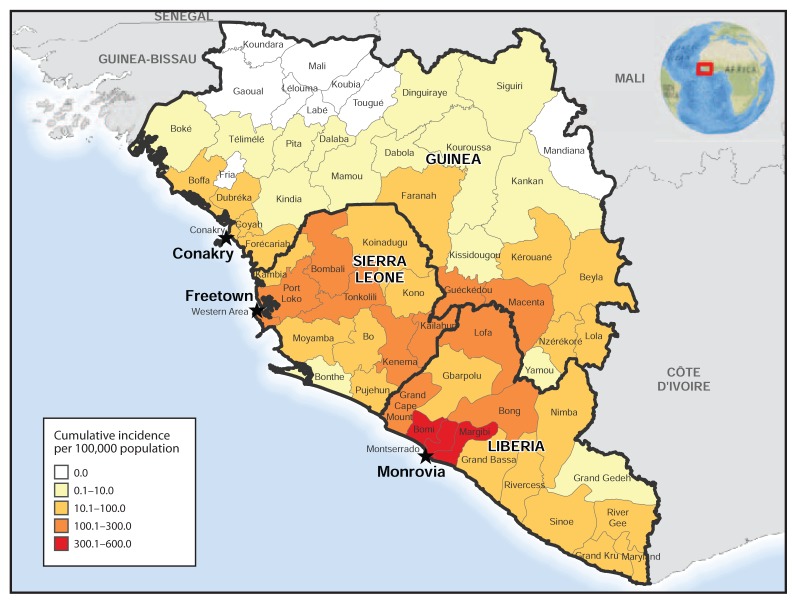
Cumulative incidence of Ebola virus disease — Guinea, Liberia, and Sierra Leone, November 30, 2014
